# Leucine Aminopeptidase to Spleen Length Ratio: A Novel Noninvasive Model for Predicting Esophageal Gastric Variceal Bleeding in Patients With Liver Cirrhosis

**DOI:** 10.1155/grp/9637489

**Published:** 2026-02-26

**Authors:** Chen Zhu, Ying Jiang, Yicun Liu, Zhaolian Bian, Lin Luo

**Affiliations:** ^1^ Department of Gastroenterology and Hepatology, Nantong Third People′s Hospital, Affiliated Nantong Hospital 3 of Nantong University, Nantong, Jiangsu, China; ^2^ School of Pharmacy, Nantong University, Nantong, Jiangsu, China, ntu.edu.cn

**Keywords:** esophageal gastric variceal bleeding (EGVB), leucine aminopeptidase (LAP)/spleen length ratio (LSR), liver cirrhosis

## Abstract

**Background:**

Esophageal gastric variceal bleeding (EGVB) is a common complication of liver cirrhosis. However, esophagogastroduodenoscopy (EGD) for detecting venous pressure is invasive, limiting its clinical application. To reduce unnecessary EGD screening, here we screened multiple commonly used noninvasive examination indicators and evaluated their potential for predicting the risk of EGVB in patients with cirrhosis.

**Methods:**

Patients with liver cirrhosis who were diagnosed with esophageal gastric varices (EGVs) for the first time were divided into groups with and without bleeding. All patients underwent serum biochemical and hematological tests, EGD, and splenic ultrasound examination. The relationships among these indices and EGVB were analyzed using logistic regression models and receiver operating characteristic (ROC) curves.

**Results:**

The neutrophil‐to‐leukocyte ratio (NEU%) and splenic vein diameter were identified as independent risk factors for EGVB, while total protein (TP), total biliary acid (TBA), leucine aminopeptidase (LAP), and platelet (PLT) count were protective factors. TP, TBA, NEU%, LAP/spleen length ratio (LSR), and PLT/spleen length ratio (PSR) were all significantly correlated with the maximum diameter of EGV blood vessels. Compared to their individual applications, the combination of TP and LSR demonstrated the highest diagnostic accuracy for EGVB.

**Conclusion:**

LSR and the combination application of LSR and TP could help predict bleeding events in patients with EGV.

## 1. Introduction

Esophageal gastric variceal bleeding (EGVB) is one of the main causes of death in patients with liver cirrhosis. Currently, the gold standard for diagnosing portal hypertension (PHT) is the hepatic venous pressure gradient (HVPG) [1]. The Baveno VI consensus indicates that patients with chronic liver disease have PHT (CSPH) when HVPG ≥ 10 mmHg [2]. HVPG > 12 mmHg is a high‐risk factor for the esophageal and gastric variceal (EGV) progression [3], while HVPG > 16 mmHg indicates very higher risk of bleeding [4]. In patients with acute bleeding, those with an HVPG > 20 mmHg have a poor prognosis. These patients exhibit high failure rates for endoscopic therapy, with or without hemostatic drugs, and have a high 1‐year mortality rate. Early assessment of PHT is crucial for predicting EGVB and implementing early intervention in patients with liver cirrhosis. However, due to the invasive characteristic of the hepatic venous pressure detection, most patients are unwilling to undergo endoscopic examination, which limits their clinical application. Therefore, finding noninvasive detection indicators to predict EGVB is of great significance in clinical practice.

EGVB is usually secondary to advanced liver cirrhosis, indicating a correlation between the degree of liver fibrosis and the risk of bleeding. Several blood biochemical indicators, such as leucine aminopeptidase (LAP), serum total protein (TP), gamma‐glutamyl transpeptidase (*γ*‐GGT), and platelet (PLT) count, are routinely used to evaluate the severity of hepatic cirrhosis. LAP is an important exopeptidase and a biomarker for liver diseases. When the liver is injured, serum levels of LAP change. LAP is also involved in the development of PHT [5]. It is known there is a close relationship between TP level and liver cirrhosis. By monitoring changes in liver function indicators, such as TP, the status of liver function and the progression of liver cirrhosis can be promptly understood, providing an important basis for the diagnosis and treatment of liver cirrhosis [6]. When liver injury causes obstruction of the extrahepatic or intrahepatic bile ducts, the *γ*‐GGT excretion is hindered [7], leading to its reflux into the bloodstream, resulting in abnormal serum level of *γ*‐GGT [8]. In addition, in patients with decompensated liver cirrhosis combined with PHT and splenic hyperfunction, most PLTs (50%–90%) are sequestered in the spleen and engulfed by an overactive mononuclear macrophage system, leading to a reduction in the peripheral PLT. Therefore, a decrease in PLT count can indirectly reflect the presence of PHT as well [9]. At present, the *γ*‐GGT/PLT ratio (GPR) has been proven to predict fibrosis in patients with chronic hepatitis B virus infection [10, 11], but its correlation with EGVB still needs further clarification. Some studies have suggested that the PLT/spleen length ratio (PSR) is associated with EGV in patients with liver cirrhosis and can be used as a triage test before endoscopic examination [12–15]. However, whether it is associated with EGVB risk factors remains unclear. Furthermore, as a biomarker for assessing the severity of liver diseases, LAP has a certain value in predicting the risk of bleeding or thrombosis [16], but the relationship between LAP and EGVB is not fully understood.

In this study, we analyzed imaging parameters and hematological and enzymological indicators in patients with EGV to explore their predictive value for EGVB. Our study provides insights into noninvasive markers for predicting the risk of EGVB in patients with liver cirrhosis.

## 2. Materials and Methods

### 2.1. Inclusion Criteria

A total of 207 patients with liver cirrhosis, who were first diagnosed with EGV, were enrolled in this study from December 2019 to December 2023.

The diagnosis of liver cirrhosis was based on clinical symptoms, laboratory tests, computed tomography/magnetic resonance imaging (CT/MRI) scans, or liver biopsies. The exclusion criteria were as follows [17]: (1) patients with liver cancer, malignant tumors of other organs, or other severe complications leading to splenic damage (such as lymphoma and Budd–Chiari syndrome); (2) patients who had previously undergone liver transplantation, partial hepatectomy, splenic embolization or splenectomy, transjugular intrahepatic portosystemic shunt (TIPS), or endoscopic treatment for esophageal and gastric varices; (3) patients with EGV caused by noncirrhosis; and (4) patients who required long‐term use of anti‐PLT drugs due to other diseases (such as after cardiac stent placement).

### 2.2. Data Collection

Cirrhosis was confirmed through endoscopic examination or treatment, and the data were collected retrospectively. The patients were divided into bleeding (*n* = 157) and nonbleeding (*n* = 50) groups.

All patients underwent a standardized clinical examination that included abdominal ultrasound and blood tests. Blood tests encompassed hematology (Sysmex XN‐2000 Hematology System, Illinois, United States) and biochemistry (HITACHI 7600 automatic biochemical analyzer, TKY, Japan). Data collection for each patient included age, gender, liver function tests (glutamic pyruvic transaminase [ALT], serum TP, albumin to globulin ratio [A/G], serum total bilirubin [TBIL], adenylate deaminase [ADA], *γ*‐GGT, alkaline phosphatase [ALP], total biliary acid [TBA], and LAP), blood routine tests (neutrophil granulocyte to leukocyte ratio [NEU%], lymphocyte to leukocyte ratio [LYMPH%], basophilic granulocyte to leukocyte ratio [BASO%], red blood cell [RBC], hemoglobin [Hb], hematocrit [HCT], PLT, mean corpuscular volume ratio [MCV%], and red blood cell volume distribution width [RDW‐SD]), coagulation parameters (prothrombin time [PT], thrombin time [TT], fibrinogen [Fbg], and D‐dimer), abdominal ultrasound, and esophagogastroduodenoscopy (EGD) examination.

### 2.3. PSR and LSR (LAP‐to‐Spleen Length Ratio) Calculation

PSR and LSR were calculated as follows:

PSR = PLT count (10^9^/L)/oblique diameter of the spleen (mm) [18].

LSR = LAP (U/L)/oblique diameter of the spleen (mm).

### 2.4. Statistical Analysis

A database of all data was established in Excel 2013. Data are expressed as the mean ± standard deviation (SD) and were statistically analyzed using SPSS software (Version 25.0) and GraphPad Prism 8 software. Normally distributed data were compared using *t*‐test between two groups, skewed distribution measurement data were expressed as median (lower quartile, upper quartile), and count data were expressed as percentages. Categorical variables and correlations were determined using Pearson′s chi‐square test or Pearson′s correlation analysis. A *p* value < 0.05 was considered statistically significant.

For variables with statistically significant differences in univariate analysis, receiver operating characteristic (ROC) curves were plotted and the areas under the curves (AUCs) were calculated to determine the optimal cutoff points on the ROC curves, which correspond to the greatest sum of the sensitivity and specificity, and to calculate the diagnostic accuracy rate. The level of significance was set at *α* = 0.05, and differences were considered statistically significant at *p* < 0.05.

### 2.5. Ethics Statement

The present study protocol was reviewed and approved by the Ethics Committee of Nantong Third People′s Hospital and Affiliated Nantong Hospital 3 of Nantong University (Approval No. EL201902). Informed consent was submitted by all subjects when they were enrolled.

## 3. Results

### 3.1. Basic Characteristics of Patients With EGV

A total of 207 patients with EGV were included in this study: 120 males (58.0%) and 87 females (42.0%). The mean age of the patients was 57.29 ± 11.06 years, ranging from 29 to 81 years. Among them, 157 patients (95 males and 62 females) were included in the bleeding group. The nonbleeding group consisted of 50 patients (25 males and 25 females). Male patients were more common in the bleeding group (> 50%), but there were no statistically significant differences between the two groups in terms of gender and age (*χ* ^2^ = 1.383, *p* = 0.240; *t* = 1.869, *p* = 0.063) (Table 1).

**Table 1:** Univariate analysis of various indices for the bleeding group and the nonbleeding group.

	Characteristics	Bleeding group (*n* = 157)	Nonbleeding group (*n* = 50)	*p*
Basic characteristics	Age	56.48 ± 11.04	59.82 ± 10.81	0.063
	Gender (male) *n* (%)	95 (60.5%)	25 (50.0%)	0.190
Serum biochemical characteristics	ALT (U/L)	23.00 (16.50, 33.00)	34.50 (22.75, 45.50)	**0.0264**
	TP (g/L)	55.40 ± 8.025	63.08 ± 7.783	**< 0.0001**
	A/G	1.48 ± 1.021	1.13 ± 0.3268	**0.0239**
	TBIL (*μ*mol/L)	22.70 (15.25, 33.30)	26.35 (17.68, 39.18)	**0.0290**
	ADA (U/L)	17.69 ± 6.910	23.29 ± 8.259	**< 0.0001**
	GGT (U//L)	27.50 (18.00, 43.00)	53.00 (28.00, 91.00)	**< 0.0001**
	ALP (U//L)	62.00 (59.50, 86.00)	104.00 (78.50, 126.00)	**< 0.0001**
	TBA (*μ*mol/L)	6.00 (1.90, 13.15)	17.15 (11.68, 44.48)	**< 0.0001**
	LAP (U//L)	27.88 ± 9.727	37.72 ± 9.030	**< 0.0001**
Hematological characteristics	NEU%	68.80 (62.65, 76.95)	57.55 (52.98, 63.70)	**< 0.0001**
	LYMPH%	19.20 (13.60, 26.60)	28.45 (21.85, 32.68)	**< 0.0001**
	BASO%	0.30 (0.00, 0.40)	0.40 (0.20, 0.50)	0.0633
	RBC (×10^12^/L)	2.660 ± 0.7891	3.448 ± 0.7058	**< 0.0001**
	Hb (g/L)	75.20 ± 21.49	105.30 ± 23.93	**<0.0001**
	HCT (%)	23.57 ± 10.18	31.57 ± 6.35	**< 0.0001**
	PLT (×10^9^/L)	48.00 (35.00, 61.00)	60.50 (45.00, 82.25)	**0.0046**
	MCV%	87.07 ± 9.494	91.02 ± 7.482	**0.0111**
	RDW‐SD (fL)	51.80 (47.15, 57.00)	49.90 (46.75, 54.30)	0.6792
Coagulation parameters	PT (s)	14.75 (13.43, 17.18)	14.3 (12.5, 15.3)	0.1981
	TT (s)	18.65 (16.76, 20.18)	19.2 (18.7, 20.9)	0.1256
	Fbg (g/L)	1.58 (1.02, 2.01)	1.6 (1.36, 2.19)	0.5417
	D‐dimer (mg/L)	1.21 (0.53, 2.59)	0.49 (0.25, 0.88)	**0.0016**
Splenic characteristics (mm)	Oblique diameter of the spleen	162.60 ± 29.53	150.00 ± 23.99	**0.0085**
	Intercostal splenic thickness	55.0 (47.0, 63.0)	50.0 (45.0, 55.0)	**0.0051**
	Diameter of splenic vein	10.0 (8.0, 11.0)	9.0 (7.0, 10.0)	**0.0007**

*Note:* Categorical variables are presented as counts (percentages); normally and nonnormally distributed continuous variables are presented as mean ± SD or median (lower quartile, upper quartile), respectively. Oblique diameter, intercostal thickness, and splenic vein diameter were determined using ultrasound. All data were analyzed by *t*‐test. Values with a *p*value < 0.05 are highlighted in bold.

Abbreviations: ADA, adenylate deaminase; A/G, albumin to globulin ratio; ALP, alkaline phosphatase; ALT, glutamic pyruvic transaminase; BASO%, basophilic granulocyte to leukocyte ratio; Fbg, fibrinogen; GGT, gamma‐glutamyl transpeptidase; Hb, hemoglobin; HCT, hematocrit; LAP, leucine aminopeptidase; LYMPH%, lymphocyte to leukocyte ratio; MCV%, mean corpuscular volume ratio; NEU%, neutrophil granulocyte to leukocyte ratio; PLT, platelet count; PT, prothrombin time; RBC, red blood cell; RDW‐SD, red blood cell volume distribution width; TBA, total biliary acid; TBIL, serum total bilirubin; TP, total protein; TT, thrombin time.

### 3.2. Hematological, Biochemical, and Splenic Characteristics of Patients With EGV

There were statistically significant differences in serological indicators between the bleeding and nonbleeding groups, including biochemical tests, hematological tests, and coagulation parameters (Table 1). Compared to the nonbleeding group, the bleeding group showed significantly reduced levels of TP, ADA, GGT, TBA, LAP, BASO, RBC, Hb, HCT, PLT, and MCV, while the D‐dimer levels in the bleeding group were significantly higher than those in the nonbleeding group. There were no statistically significant differences in RDW‐SD, PT, TT, and Fbg levels between the two groups. The reason for D‐dimer elevation in patients with EGVB may relate to multiple mechanisms, including systemic coagulation–fibrinolysis activation [19, 20], impaired hepatic metabolism/clearance [21], and inflammation‐driven [22]. In addition, although the levels of ALT, A/G, ALP, and NEU% were normal, there were statistical differences between the bleeding and nonbleeding groups.

The oblique diameter, intercostal splenic thickness, and diameter of splenic vein were determined using ultrasound. The results showed that the three parameters in the bleeding group were higher than those in the nonbleeding group (Table 1).

### 3.3. Influence Factors Associated With EGVB

After comparing the biochemical, hematological, and splenic characteristics of patients in the bleeding and nonbleeding groups, binary logistic regression models were used to analyze the risk factors for EGVB (Table 2). The analysis showed that TP, TBA, LAP, NEU%, splenic vein diameter, and PLT were the independent influencing factors for EGVB. Specifically, splenic vein diameter and NEU% were identified as risk factors for bleeding in patients with EGV and liver cirrhosis, whereas TP, TBA, LAP, and PLT were protective factors.

**Table 2:** Multivariate logistic regression analysis of various indices for the bleeding group and the nonbleeding group.

	Parameter	*B*	SE	Wald	*p*	Exp (*B*)	EXP (*B*) 95% CI	
							Lower limit	Upper limit
Serum biochemical characteristics	TP	−0.148	0.042	12.423	**0.000**	0.862	0.794	0.936
	GGT	−0.003	0.008	0.100	0.752	0.997	0.982	1.013
	TBA	−0.036	0.018	4.059	**0.044**	0.965	0.932	0.999
	LAP	−0.095	0.041	5.390	**0.020**	0.910	0.840	0.985
	TBIL	−0.018	0.027	0.477	0.490	0.982	0.932	1.034
	ADA	−0.020	0.051	0. 158	0.691	0.980	0.886	1.083
	Constant	14.634	2.984	24.052	0.000	2,268,053.583		
Hematological characteristics	NEU%	0.178	0.060	8.733	**0.003**	1.195	1.062	1.344
	LYMPH%	0.060	0.067	0.802	0.370	1.062	0.931	1.211
	RBC	−1.071	1.481	0.523	0.470	0.343	0.019	6.248
	Hb	−0.017	0.045	0.135	0.713	0.983	0.900	1.075
	HCT	−0.042	0.039	1.135	0.287	0.959	0.888	1.036
	PLT	−0.022	0.010	4.357	**0.037**	0.979	0.959	0.999
	MCV	−0.072	0.072	0.993	0.319	0.931	0.808	1.072
	Constant	1.963	8.849	0.049	0.824	7.119		
Splenic characteristics	Oblique diameter of the spleen	0.000	0.009	0.001	0.975	1.000	0.981	1.018
	Intercostal splenic thickness	0.016	0.019	0.642	0.423	1.016	0.978	1.055
	Diameter of splenic vein	0.210	0.098	4.635	**0.031**	1.234	1.019	1.494
	Constant	−1.619	1.085	2.225	0.136	0.198		

*Note:* The data were screened using univariate analysis, and the related influencing factors were analyzed using binary logistic regression analysis. The data were analyzed by the Hausman test. Values with a *p*value < 0.05 are highlighted in bold.

Abbreviations: ADA, adenylate deaminase; GGT, gamma‐glutamyl transpeptidase; Hb, hemoglobin; HCT, hematocrit; LAP, leucine aminopeptidase; LYMPH%, lymphocyte to leukocyte ratio; MCV%, mean corpuscular volume ratio; NEU%, neutrophil granulocyte to leukocyte ratio; PLT, platelet; RBC, red blood cell; TBA, total biliary acid; TBIL, serum total bilirubin; TP, total protein.

### 3.4. Correlation Between the Severity of EGV and Influence Factors, PSR and LSR

Based on endoscopic examinations, the maximum diameter of the esophageal and gastric veins of each patient was determined using EGD to assess the severity of EGV. To determine whether the influencing factors of EGVB impact the maximum diameter of EGV blood vessels, linear regression models were performed to explore the relationship between the diameter and these influencing factors, including TP, TBA, NEU%, LAP, PLT, and splenic vein diameter. In Figures 1a, 1b, 1c, 1d, 1e, and 1f, the results showed that TP and TBA were negatively correlated with the maximum diameter of EGV blood vessels, whereas NEU% demonstrated a positive correlation. The PLT and diameters of splenic vein were unrelated to the maximum diameter of the EGV blood vessels. Although LAP showed a negative trend in relation to the maximum diameter of the EGV blood vessels, this association did not reach statistical significance, possibly due to the partial absence of LAP data. These findings suggested that TP, TBA, and NEU% have a notable correlation with the maximum diameter of EGV blood vessels, implying their potential as candidate diagnostic indicators for predicting EGVB.

Figure 1Correlation between the maximum diameter of esophageal gastric vessels and influence factors in patients. Linear regression models were performed on the relationships between the maximum diameter of EGV blood vessels and the following influence factors: (a) TP, (b) TBA, (c) NEU%, (d) LAP, (e) PLT, (f) the diameter of splenic vein, (g) LSR, and (h) PSR. Abbreviations: TP, total protein; TBA, total biliary acid; LAP, leucine aminopeptidase; NEU%, neutrophil granulocyte to leukocyte ratio; PLT, platelet; LSR = LAP (U/L)/oblique diameter of the spleen (mm); PSR = platelet count (10^9^/L)/oblique diameter of the spleen (mm).

**Figure:**
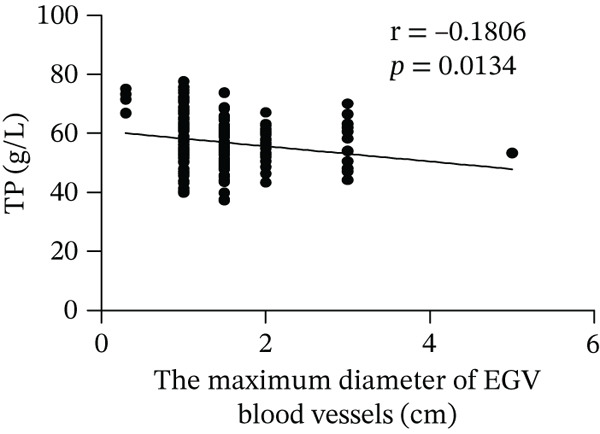
(a)

**Figure:**
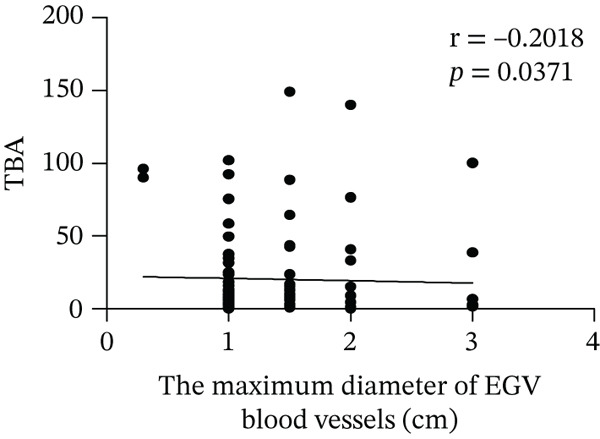
(b)

**Figure:**
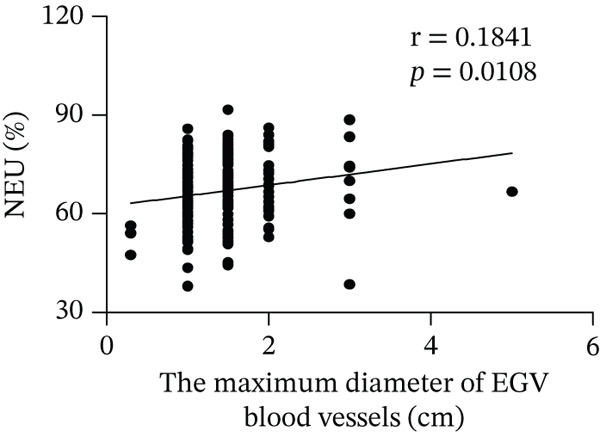
(c)

**Figure:**
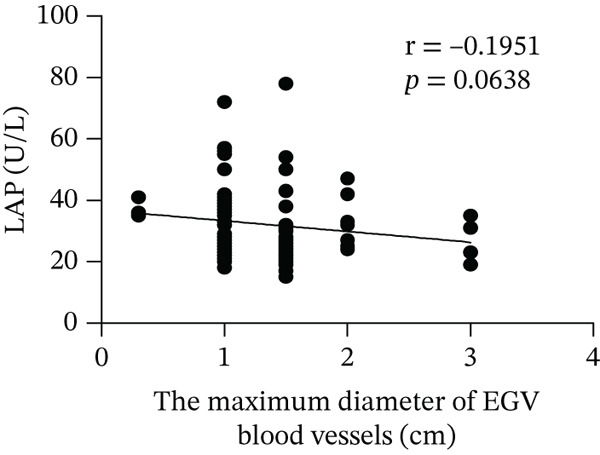
(d)

**Figure:**
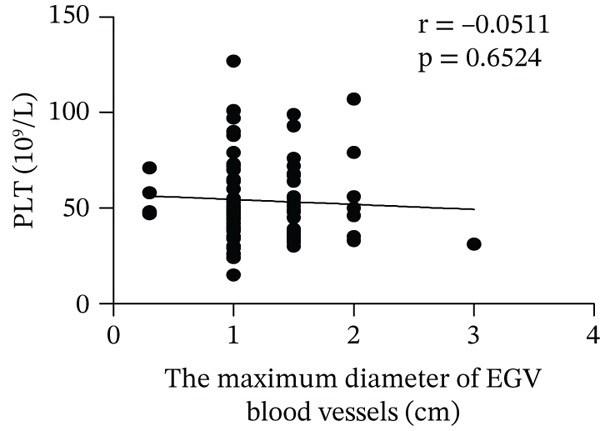
(e)

**Figure:**
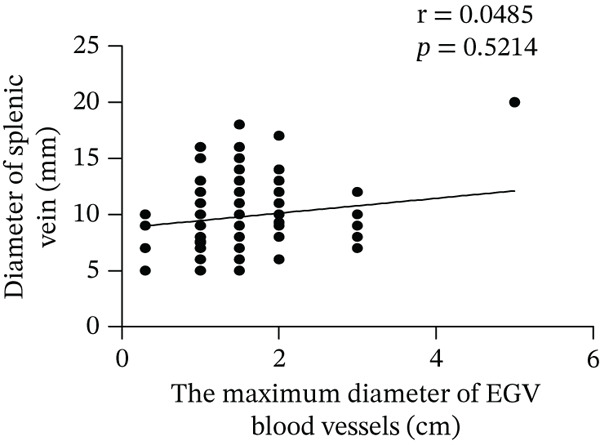
(f)

**Figure:**
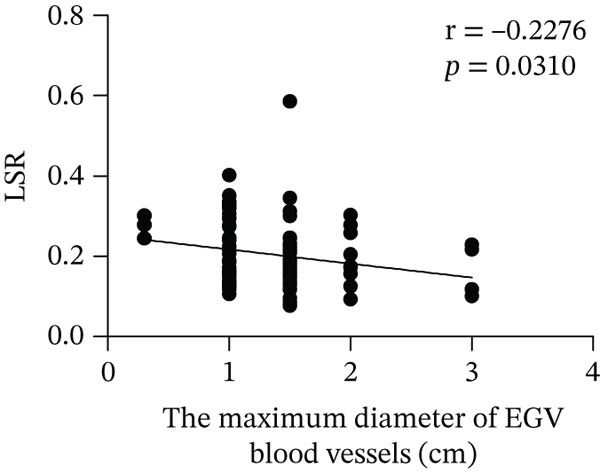
(g)

**Figure:**
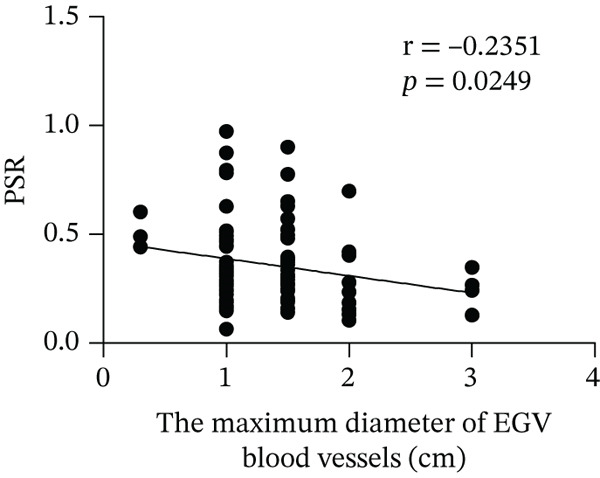
(h)

Given that the severity of EGV is influenced by both the maximum diameter of the EGV blood vessels and the extent of hypersplenism following cirrhosis, assessing the relationship between PSR and LSR to EGVB may provide valuable insights. The data in Table 3 show that both PSR and LSR decreased in the bleeding group compared to the nonbleeding group of patients with EGV. To further explore these associations, linear regression models were employed to analyze the relationship between the maximum diameters of the esophageal and gastric veins and LSR or PSR. The results revealed a negative correlation between the maximum vein diameter and LSR or PSR (Figure 1g,h). This suggests that patients with lower LSR or PSR values tend to have larger maximum diameters of the esophageal and gastric veins, which correlates with a higher risk of bleeding. Thus, the LSR and PSR have the potential to serve as candidate diagnostic indicators for predicting EGVB.

**Table 3:** Univariate analysis of the combined ratio indices for the bleeding group and the nonbleeding group.

Characteristics	Bleeding group (*n* = 157)	Nonbleeding group (*n* = 50)	*p*
PSR (×10^9^/L/mm)	0.2882 (0.1968, 0.3708)	0.4436 (0.3504, 0.5714)	**< 0.0001**
LSR (×U/L/mm)	0.1645 (0.1301, 0.2252)	0.3007 (0.2062, 0.3268)	**< 0.0001**

*Note:* Nonnormally distributed continuous variables were presented as median (lower and upper quartiles). Data were analyzed by *t*‐test. Values with a *p*value < 0.05 are highlighted in bold.

Abbreviations: LSR, leucine aminopeptidase (U/L)/oblique diameter of the spleen (mm); PSR, platelet count (10^9^/L)/oblique diameter of the spleen (mm).

### 3.5. The Diagnostic Value of Influence Factors, LSR and PSR in Predicting Bleeding Risk in EGV Patients

To ascertain the optimal sensitivity and specificity cutoff values for candidate diagnostic indicators in predicting EGVB and to evaluate the efficacy of the models, the area under the ROC curve (AUROC) of each parameter was calculated [23]. Models with an AUC value exceeding 0.7 were considered useful, while an AUC value ranging from 0.8 to 0.9 indicated excellent diagnostic accuracy [24]. The AUROC values for the TP, TBA, LAP, and LSR score all surpassed 0.80, higher than NEU% and PSR (Figures 2a, 2b, 2c, 2d, 2e, and 2f). Multivariate logistic analysis was used to predict EGVB (Figure 2g,h and Table 4). The data showed that both the AUCs of the combinations of LSR with TP and LSR with TBA were greater than 0.87, and the combination of TP and LSR exhibited the highest diagnostic sensitivity, compared to the individual application of TP, TBA, or LSR.

Figure 2Receiver operating characteristic curves for predicting the various influencing factors of bleeding risk in patients with esophageal gastric varices. For variables with statistically significant differences in univariate analysis, receiver operating characteristic curves were plotted and the areas under the curves were calculated to determine the optimal cutoff points on the curves corresponding to the greatest sum of the sensitivity and specificity and to calculate the rate of diagnostic accuracy. The ROC curves are depicted for (a) TP, (b) NEU%, (c) TBA, (d) LAP, (e) LSR, and (f) PSR. The multivariate logistic analysis was used to fit the ROC curves for (g) the combination of LSR with TP and (h) the combination of LSR with TBA in predicting bleeding risk in EVG patients. The red dashed line represents the reference line. Abbreviations: AUCs, areas under the curve; TP, total protein; TBA, total biliary acid; LAP, leucine aminopeptidase; NEU%, neutrophil granulocyte to leukocyte ratio; LSR = LAP (U/L)/oblique diameter of the spleen (mm); PSR = platelet count (10^9^/L)/oblique diameter of the spleen (mm).

**Figure:**
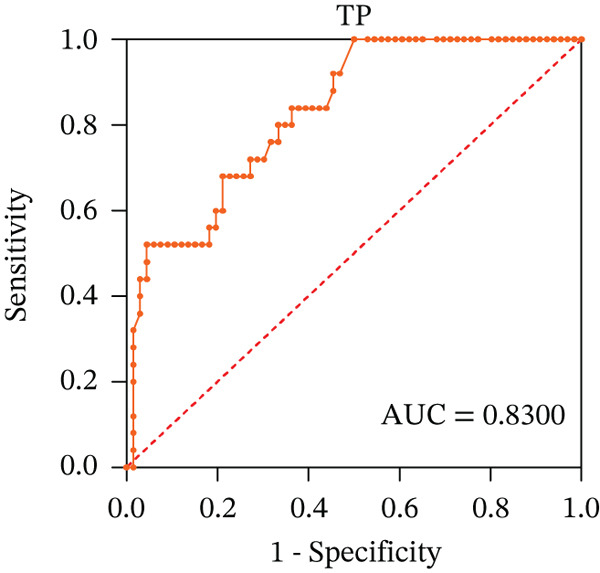
(a)

**Figure:**
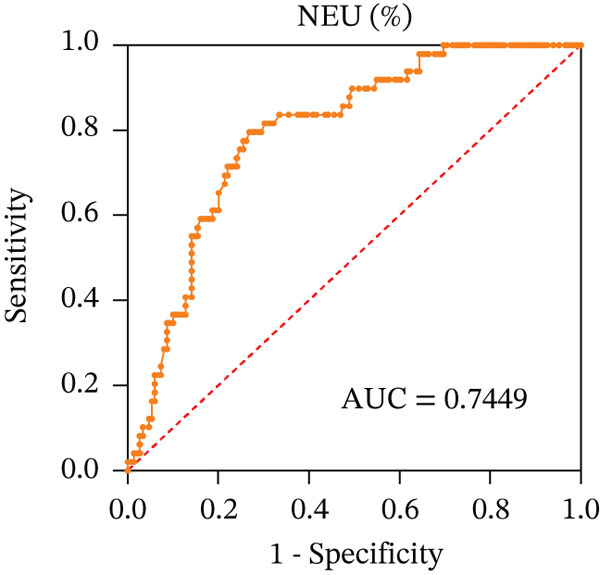
(b)

**Figure:**
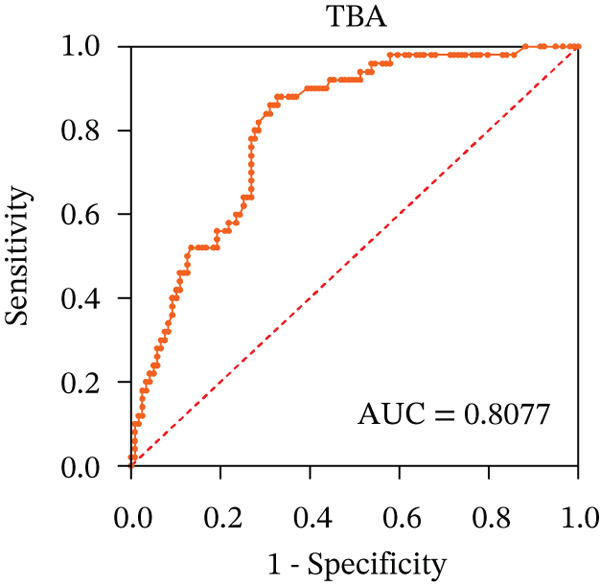
(c)

**Figure:**
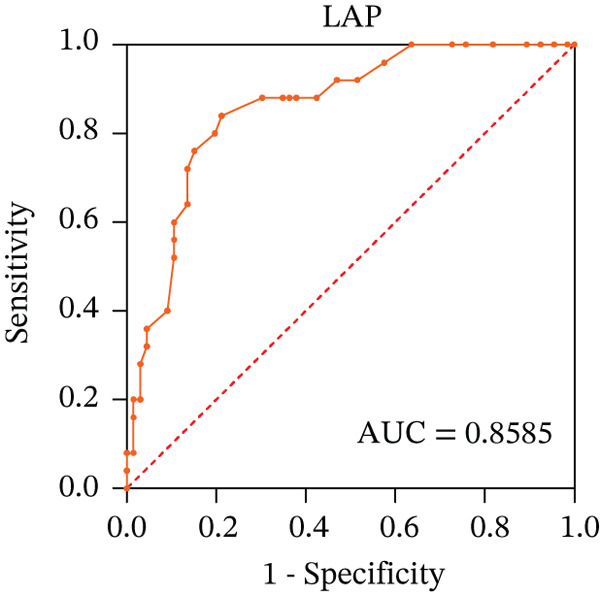
(d)

**Figure:**
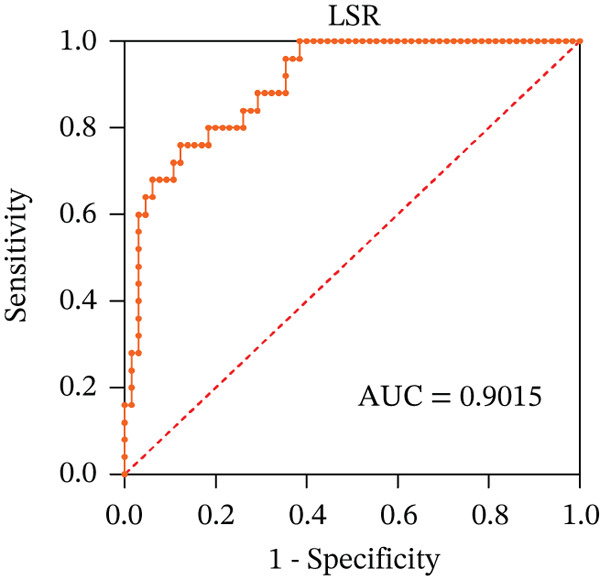
(e)

**Figure:**
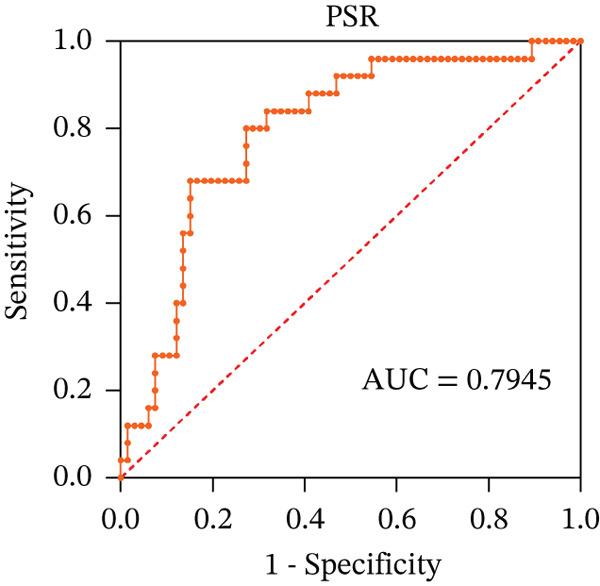
(f)

**Figure:**
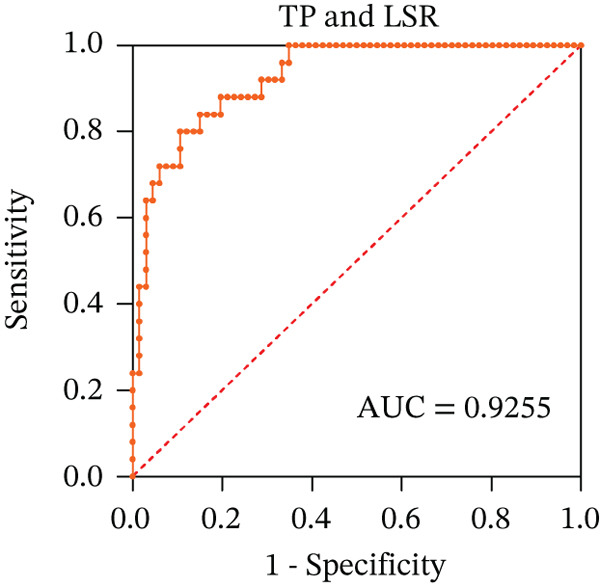
(g)

**Figure:**
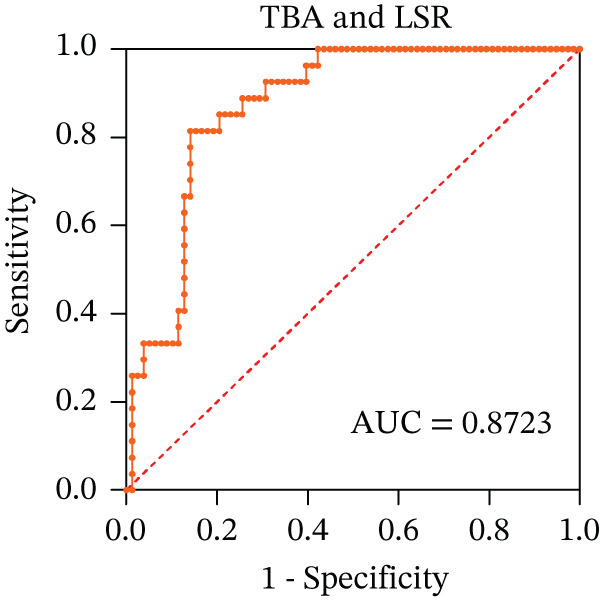
(h)

**Table 4:** Predictive value of multi‐index combination for the risk of esophageal gastric variceal bleeding.

Variables	TP	LSR	TBA	Combined index	
				TP and LSR	TBA and LSR
AUC	0.8300	0.9015	0.8077	0.9255	0.8723
Cutoff value	56.8000	0.2427	9.3000	0.6233	0.7137
Sensitivity (%) (95% CI)	84.00 (63.35–93.60)	76.00 (56.57–88.50)	88.00 (76.20–94.38)	80.00 (60.87–91.14)	81.48 (63.30–91.82)
Specificity (%) (95% CI)	63.64 (51.58–74.19)	87.69 (77.55–93.63)	67.23 (58.37–75.01)	89.39 (79.69–94.77)	85.90 (76.49–91.94)
Youden index	2.3100	0.6369	0.5523	0.6939	0.6738
*p* value	**< 0.0001**	**< 0.0001**	**< 0.0001**	**< 0.0001**	**< 0.0001**

*Note:* The sensitivity and specificity for all patients were predicted based on ROC curve analysis, and Youden′s index was calculated to determine the cutoff value. Values with a *p*value < 0.05 are highlighted in bold.

Abbreviations: AUC, area under the curve; LSR, leucine aminopeptidase (U/L)/oblique diameter of the spleen (mm); TBA, total biliary acid; TP, total protein.

## 4. Discussion

Liver cirrhosis is a repair response following injury in patients with chronic liver damage [25], which can lead to severe complications such as PHT, EGVB, sepsis, hepatorenal syndrome, and hepatic encephalopathy [26, 27]. The incidence of EGVB among patients with PHT ranges from 17% to 57% [28] and is one of the most common causes of death in patients with liver cirrhosis. Epidemiological investigation has found that the annual incidence of EGV development in patients with liver cirrhosis is approximately 7% [29], with a cumulative 5‐year incidence of 21% [30]. Therefore, it is currently recommended that all patients with chronic liver disease diagnosed with liver cirrhosis undergo EGV screening using upper gastrointestinal endoscopy (UGIE) [31]. Follow‐up endoscopies are then conducted at intervals of 1–3 years, based on the initial EGV grade and the progression of chronic liver disease [32]. However, the utilization of UGIE for EGV screening is limited by factors such as cost, invasiveness, and patient discomfort [33]. A recent survey indicated that approximately half of the patients did not undergo EGV screening [34]. In addition, there are significant interobserver variations in the assessment of EGV classification, making UGIE an imperfect gold standard [35]. Given that EGV is an independent risk factor for mortality, there is an urgent need to improve the prediction and diagnosis of this condition [36].

To avoid unnecessary endoscopy for low‐risk patients and ease the growing burden on endoscopy units, several studies have demonstrated that certain biochemical, clinical, and ultrasonographic parameters, either alone or in combination, possess good predictive power [37] for the noninvasive assessment of EGVB [38]. Therefore, the identification of ideal and easily accessible noninvasive markers to evaluate the risk of EGVB, and reduce the number of endoscopic procedures needed for screening and managing EGV in patients with liver cirrhosis, is of paramount clinical importance. ‌In this work, we found that two straightforward indices, TP and LSR, which are composed of three readily available laboratory test results (TP, LAP, and spleen longitudinal diameter), could accurately predict the bleeding risk of EGV patients with liver cirrhosis.

TP, the most abundant substance in the solid components of serum, is commonly used with albumin, globulin, and the albumin/globulin ratio to comprehensively evaluate liver function and the severity of liver cirrhosis [39]. Our data showed that TP serves as an independent risk factor for EGVB and has a strong linear correlation with the maximum diameter of the EGV blood vessels obtained from EGD. In addition, the AUC for TP in identifying high‐risk EGVB patients was excellent.

LAP, another biomarker for assessing liver disease severity, may contribute to PHT by affecting liver metabolism and immune function, thereby serving as an indicator to evaluate the risk of EGVB [16]. Although LAP is less commonly used clinically than AST, ALT, and GGT, univariate analysis and multivariate logistic regression analyses in this study demonstrated its higher specificity in EGVB evaluation. However, no statistical significance relationship was observed between LAP and the maximum diameter of the EGV blood vessels, suggesting that LAP may be best used as part of a comprehensive assessment along with other biomarkers and clinical manifestations. Therefore, we employed LSR to evaluate bleeding risk in patients with EGV. Our results showed LSR was lower in the bleeding group than in the nonbleeding group. A strong linear correlation was found between LSR and the maximum diameter of the EGV blood vessels, and the AUC for identifying high‐risk EGVB patients was notably high (AUC = 0.9015). To date, no clear evidence exists for a direct association between LAP and splenic diameter, but we propose that combining LAP and splenic diameter can enhance the prediction of EGVB. The rationale is as follows: (1) Liver cirrhosis leads to increased portal pressure, causing PHT, which results in hypersplenism and splenic diameter enlargement. Colli et al. reported that splenic length is associated with bleeding risk [40]. (2) Liver cirrhosis and its complications (e.g., cholestasis) can directly elevate serum LAP levels. Therefore, in patients with liver cirrhosis, increased splenic diameter, abnormal PLT levels, and elevated serum LAP may coexist. In this context, both LAP and splenic diameter reflect the severity of the underlying liver disease and may exhibit a “concomitant” or “correlative” relationship. Additionally, PHT can be complicated by upper gastrointestinal bleeding, and recent literature has reported an association between serum LAP levels and the risk of bleeding or thrombosis [16]. Given that this study focuses on bleeding events resulting from the progression of decompensated liver cirrhosis, which directly affects PLT, we employed the LAP instead of PLT.

In 2003, Giannini et al. published a seminal report demonstrating that PSR could identify patients with esophageal varices (EVs) with a high degree of accuracy [41]. However, subsequent studies have yielded inconsistent results regarding PSR′s efficacy in EV detection. Ying et al. found that PSR had a pooled sensitivity of 94% and a specificity of 84%, suggesting that it is a valuable noninvasive screening tool for EV [42]. A meta‐analysis by Chen et al. revealed that ROC for PSR in identifying varices and high‐risk varices was 0.8719 and 0.8132, respectively, with sensitivities of 0.84 and 0.78 [43]. In the present study, we investigated various biomarkers to determine their ability to predict EGVB. Although PLT was an independent risk factor for bleeding in patients with EGV, the linear correlation between the maximum diameter of EGV blood vessels and PLT was not statistically significant (*p* > 0.05). PSR showed a good linear correlation with the maximum diameter of EGV blood vessels, but its AUC for identifying high‐risk EGVB was only 0.7945. Additionally, TBA and NEU% showed good linear correlations with the maximum diameter of EGV blood vessels; however, their AUC values were approximately 0.80. We also evaluated the predictive value of GPR for EGVB but found no statistically significant correlation between GPR and the maximum diameter of EGV blood vessels (*p* = 0.3967) (results not shown). These findings indicate that none of the aforementioned indicators is ideal for predicting EGVB.

In the present work, our initial research findings indicate that the composite parameter LSR is highly effective in distinguishing between patients with EGV and EGVB, suggesting its clinical utility. First, from a statistical perspective, LSR demonstrated a linear correlation with the maximum diameter of EGV blood vessels associated with EGVB. This correlation underscores the potential of LSR as a valuable indicator for assessing bleeding risk. Second, from a clinical perspective, relying solely on parameters such as TP, TBA, LAP, and spleen length can be misleading when predicting EGVB in patients with chronic liver disease. These parameters can fluctuate due to various factors and cannot be attributed exclusively to PHT, which is the main contributor to EGVB. Third, our statistical analysis validated the differences in indicators between the bleeding and nonbleeding groups, specifically focusing on TP and LSR, both of which are included in routine examinations for clinically diagnosed EGVB patients. This combined approach, integrating LSR with other relevant parameters, offers several advantages: low‐risk, highly accurate, simplicity, cost‐effectiveness, and ease of follow‐up. Our data demonstrate that the combined application of TP and LSR can be regarded as a highly promising first‐line indicator for estimating EGVB risk, especially in regions with limited medical resources. Given these benefits, the combined application method should be actively explored and promoted in both clinical practice and scientific research‌.

Additionally, the applicability of our findings to community‐based practices remains to be determined. Although TP and LSR are straightforward and precise, overlap may occur in patients with different stages of fibrosis. Therefore, the utilization of these two scoring indicators to predict the bleeding risk in patients with EGV must be confirmed through prospective studies.

## 5. Conclusion

In the present study, we found that TP and LAP are independent risk factors for bleeding in patients with EGV and liver cirrhosis. The LSR model exhibits superior predictive and diagnostic values for EGVB compared to most noninvasive models. Furthermore, the combination of TP and LSR was more effective in predicting bleeding risk in EVG patients than using either parameter alone, indicating its potential as a supplementary tool for the endoscopic screening of EGVB‌‌.

## Author Contributions

Conceptualization: Chen Zhu and Lin Luo. Data curation: Chen Zhu and Ying Jiang. Formal analysis: Ying Jiang. Funding acquisition: Zhaolian Bian. Investigation: Chen Zhu, Yicun Liu, and Zhaolian Bian. Methodology: Ying Jiang and Lin Luo. Writing—original draft: Ying Jiang. Writing—review and editing: Lin Luo. Chen Zhu and Ying Jiang contributed equally.

## Funding

This study was supported by the Science and Technology Project of Nantong (JC2023115).

## Conflicts of Interest

The authors declare no conflicts of interest.

## Data Availability

Data available on request from the authors.
